# Time-of-Day-Associated Variation in Serum CO_2_ Patient-Result Distribution and Its Impact on PBRTQC Performance

**DOI:** 10.3390/diagnostics16213504

**Published:** 2026-10-26

**Authors:** Ziqing Jiang, Xuemei Wei, Lei Lin, Jie Feng, Yanhong Gao

**Affiliations:** Department of Clinical Laboratory, The First Medical Center of Chinese PLA General Hospital, Beijing 100853, China; ziqing.jiang@outlook.com (Z.J.); weixuemei301@163.com (X.W.); linlei_research@163.com (L.L.)

**Keywords:** patient-based real-time quality control (PBRTQC), serum carbon dioxide, time-of-day-associated variation, time-stratified quality control, acidosis-to-normal reclassification

## Abstract

**Background:** Patient-based real-time quality control (PBRTQC) is now widely used in clinical labs, yet the impact of time-dependent variation on its performance remains understudied, particularly with regard to serum carbon dioxide (CO_2_). This study explores how time-of-day-associated variation in the serum CO_2_ patient-result distribution affects PBRTQC outcomes and clinical acidosis classification, and an optimization strategy is evaluated. **Methods:** We analyzed 168,957 serum CO_2_ results collected over one year (2025) using three biochemical analyzers. Moving average-based PBRTQC was compared between peak (10:00–13:00) and off-peak periods, using either conventional unified limits or time-stratified separate limits. **Results:** Serum CO_2_ patient-result distribution shifted significantly toward higher values during the peak period (23.6 vs. 23.0 mmol/L, *p* < 0.001). A unified control strategy led to a marked rise in false-positive rates (0.955% vs. 0.062%). In a simulated category-transition analysis, 92 of 1212 samples (7.59%) were reclassified, of which 88 were acidosis-to-normal shifts. The time-stratified model reduced peak false positives to 0.069% while maintaining efficient detection of error. **Conclusions:** Time-of-day-associated variation in the distribution of patients’ serum CO_2_ impairs PBRTQC specificity during peak hours. Time-stratified control limits reduce false-positive rates while maintaining error detection, offering a practical solution for reliable serum CO_2_ monitoring. These findings should be interpreted as descriptive, given the cross-sectional design and the potential influence of patient case mix.

## 1. Introduction

Serum CO_2_ is a key indicator of the acid–base status in patients with respiratory and metabolic diseases. It is routinely used in the evaluation of metabolic acidosis, respiratory alkalosis, and mixed acid–base disturbances. In emergency and intensive care settings, CO_2_ values are often used to support clinical decision-making [1].

Patient-based real-time quality control (PBRTQC) is an emerging form of internal quality control (IQC) that continuously monitors analytical performance using actual patient sample data, complementing traditional commercial control materials. The three commonly used PBRTQC algorithms are moving average (MA), exponentially weighted moving average (EWMA), and moving median (MM). The MA method is the simplest and most widely used algorithm; it calculates the average of a fixed number of consecutive patient results to smooth out random fluctuations and detect systematic changes in the analytical method. EWMA is more sensitive to recent changes and responds quickly to single shifts, while MM is more robust to extreme values [2]. In this study, MA [3,4,5,6] was chosen as the primary PBRTQC algorithm due to its simplicity, widespread clinical use, and stability for routine laboratory operations.

PBRTQC performance may be influenced by time-of-day-associated variation in patient-result distributions, which can arise from multiple sources. These include patient/case-mix variation (e.g., differences in disease severity or clinical department of origin across time periods), preanalytical effects (e.g., sample transport time, collection tube type, or tourniquet duration), workflow/sample-transport effects (e.g., batch processing or centrifugation delays), environmental effects (e.g., fluctuations in indoor pCO_2_), and true within-person biological variation [2]. Although it has been shown that the serum CO_2_ patient-result distribution varies with time of day [7], its effect on PBRTQC performance has not been systematically studied or quantified. Whether time-of-day-associated variation in patient-result distribution affects the false-positive rate (FPR) and error detection sensitivity of PBRTQC or leads to potential misclassification of acidosis has not been determined yet [8].

Therefore, the aims of this paper are to (1) describe the time-of-day-associated variation in serum CO_2_ patient-result distribution across different daytime periods under routine clinical conditions, (2) investigate whether this variation affects PBRTQC performance indicators, including FPR and error detection efficiency, and (3) conduct an exploratory analysis of potential acidosis misclassification using a simulated category-transition approach [9]. Given the retrospective cross-sectional design, the present study cannot distinguish among the mechanisms listed above; the results should therefore be interpreted as descriptive of real-world PBRTQC behavior rather than as evidence of a physiological diurnal rhythm.

## 2. Methods

### 2.1. Study Design and Data Source

This retrospective study was performed at the First Medical Center of the Chinese People’s Liberation Army General Hospital. The study was approved by the Institutional Review Board (S2024-596-01), and informed consent was waived for de-identified routine laboratory data. Serum CO_2_ results from 1 January to 31 December 2025 were extracted from the laboratory information system (LIS). We included 168,957 samples from patients aged 0–104 years (mean 56.90, SD 19.290) who visited the emergency department. All tests were conducted on three Roche cobas c-series analyzers (C8K4, C8K5, C8K11). Measurements used the Roche cobas CO_2_ assay (cat. no. 06407102188), an enzymatic colorimetric method with PEPC. Calibration and quality control followed the manufacturer’s protocol using relevant calibrator (cat. no. 20751995) and Precipath U plus controls (cat. nos. 12149435, 12149443). The analytical measurement interval (AMI) was 2–50 mmol/L, with within-run and between-run CVs ≤ 1.0% and ≤2.3%, respectively.

Results outside the AMI (2–50 mmol/L) were considered analytically invalid. In addition, results < 10 or >40 mmol/L were excluded to remove implausible physiological values, in line with previous PBRTQC studies. These study-specific thresholds are narrower than the AMI and were applied to ensure that the analyzed dataset reflected clinically plausible serum CO_2_ results. No further exclusion criteria were used. A flow diagram of sample inclusion and exclusion is provided in Figure S1.

Among 80,318 unique patients, 535 (0.67%) had at least one day with repeated testing, contributing 1189 records (0.70% of all measurements). Because repeated measurements from the same patient were available for less than 1% of records, a within-person paired analysis of time-of-day variation was not feasible. The present results therefore describe differences between patient populations presenting at different times of day, not within-individual temporal changes.

### 2.2. Definition of Peak and Off-Peak Periods

We determined peak and off-peak time windows empirically according to hourly profiles of the serum CO_2_ patient-result distribution. The peak interval was set at 10:00–13:00, the interval with the highest observed hourly mean (mean difference 0.60 mmol/L, 95% CI 0.58–0.62, *p* < 0.001). The off-peak interval included 8:00–9:00 and 14:00–17:00.

While the two off-peak segments showed a small statistical difference (0.17 mmol/L, *p* < 0.001), this difference was clinically unimportant and well below the 22 mmol/L cutoff for acidosis. We therefore combined these periods to strengthen study robustness.

The peak interval was selected from the same dataset used for subsequent PBRTQC evaluation. This introduces a risk of overfitting, and the reported performance differences should be interpreted as descriptive rather than confirmatory. We did not perform an independent derivation/validation split because the primary aim was to describe real-world PBRTQC behavior under routine conditions. This limitation is acknowledged in the Discussion.

### 2.3. PBRTQC Parameter Settings

We adopted the moving average (MA) method for PBRTQC due to its simplicity and suitability for daily laboratory use [3,5]. Parameters were chosen as follows: block size of 15 to balance sensitivity and stability [10]; control limits fixed at ±1.2 SD of the overall/annual mean to minimize false alerts; and data trimmed at the 5th and 95th percentiles before analysis [10,11]. All calculations were run separately for each instrument and each time period. The MA was computed sequentially within each period, and an alert was recorded when the MA exceeded the control limits.

### 2.4. False-Positive Rate (FPR) Calculation

The FPR was calculated using error-free historical data. For each analyzer and period, the MA was computed sequentially. An alert was recorded if the MA exceeded control limits. FPR was defined as the percentage of MA blocks that triggered an alert. The numbers of MA blocks per period and per analyzer are listed in Table S4.

### 2.5. Error Simulation and ANPed Calculation

To assess error detection capability, graded constant positive biases (5%, 8%, 10%, 12%) were inserted at random start points. The 12% bias, equivalent to the allowable total error (TEa) of serum CO_2_, was set as the primary endpoint owing to its clinical impact on patient management [11,12]. Outcomes of other bias levels are presented in the Supplementary Materials.

ANPed and Ped were calculated for each scenario, with 10 independent simulation runs conducted per condition. The simulation parameters are described in detail in the Methods, and the simulation code is available from the corresponding author upon request.

### 2.6. Simulated Category-Transition Analysis

An exploratory analysis was performed to examine the potential clinical impact of time-of-day-associated variation in the serum CO_2_ patient-result distribution. Patients were stratified into three categories: acidosis (CO_2_ < 22 mmol/L), normal (22–29 mmol/L), and alkalosis (CO_2_ > 29 mmol/L). The analysis was restricted to emergency resuscitation room samples collected during off-peak periods in 2025 (*n* = 1212), which served as the reference classification. This subset was selected because it represents a clinically relevant population in which acid–base status is frequently monitored, and because its off-peak CO_2_ distribution (mean 19.8 mmol/L) reflects a high prevalence of acidosis. Since individual paired measurements were not available in this retrospective cross-sectional dataset, peak-period values were simulated by adding the observed mean shift of 0.72 mmol/L to off-peak results. This shift was derived from the emergency resuscitation room subset itself (peak mean 20.5 vs. off-peak mean 19.8 mmol/L) rather than from the full dataset (0.60 mmol/L), because the primary analysis of interest was the clinical classification shift within this high-acuity population. The simulated category-transition matrix was then constructed by cross-tabulating the reference (off-peak) and simulated (peak) classifications. Given the simulated nature of this analysis and the lack of paired longitudinal data, these results should be interpreted as exploratory and hypothesis-generating, rather than confirmatory.

### 2.7. Statistical Analysis

Welch’s *t*-test was used for between-group comparisons of continuous variables, given heterogeneous variance (Levene’s test, *p* < 0.001). Effect size was expressed as Cohen’s d. Given the large sample size (*n* = 168,957), extremely small differences can produce *p* < 0.001; therefore, effect sizes and 95% confidence intervals were emphasized over statistical significance. A two-sided *p* < 0.05 was defined as statistically significant. All statistical tests were conducted with R software (version 4.5.3).

## 3. Results

### 3.1. Time-Dependent Distribution of Serum CO_2_ Concentration

**Figure 1:**
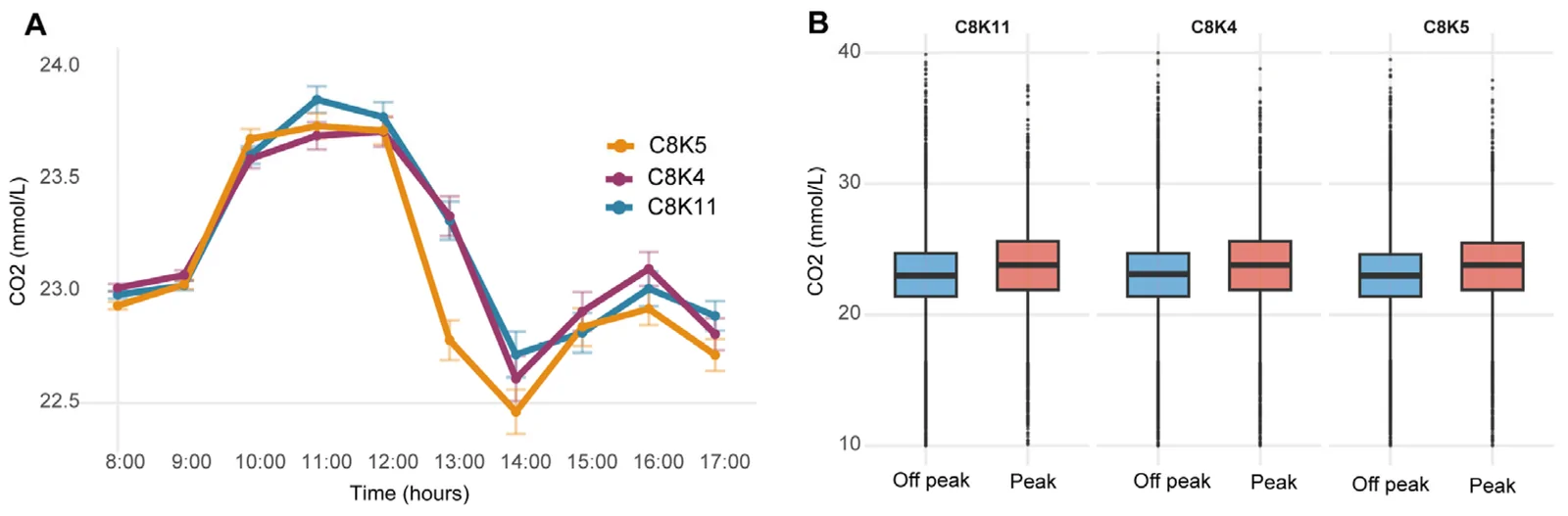
(**A**) Time-series plot of hourly mean CO_2_ levels (±standard error). Gray shading and red symbols indicate the peak period (10:00–13:00). (**B**) Hourly boxplot of CO_2_ distribution. Boxes represent IQR, middle line shows median, whiskers extend to 1.5× IQR. Gray shading and red boxes denote peak values.

A total of 168,957 serum CO_2_ results were used for the final analysis. As shown in Figure 1A, the serum CO_2_ patient-result distribution varied across daytime hours, with the hourly mean lowest before 10:00 and highest between 11:00 and 13:00. The mean of the patient-result distribution at peak was 23.6 mmol/L, and at off-peak it was 23.0 mmol/L; the difference was 0.60 mmol/L (95% CI 0.58–0.62), and this difference was statistically significant (*p* < 0.001). This time-of-day-associated pattern was consistent across all three analyzers (Figure 1B and Table S1), and detailed values by instrument and period are provided in Table S1.

To examine whether the observed time-of-day-associated variation could be explained by differences in patient composition, we compared the distribution of clinical department, sex, and age between peak and off-peak periods. The department distribution differed markedly between periods (χ^2^ = 34,133, df = 3, *p* < 2.2× 10^−16^. Ward samples accounted for 76.42% of off-peak records but only 29.24% of peak records, whereas emergency department samples increased from 9.46% off-peak to 30.99% peak, and outpatient samples increased from 8.30% to 37.10% (Table 1). The sex distribution also differed significantly (χ^2^ = 53.61, df = 1, *p* = 2.445× 10^−13^), with males comprising 56.0% of off-peak and 58.2% of peak samples (Table S3). Age-stratified analysis showed that the peak-period mean was significantly higher than the off-peak mean in all age groups except <18 years (18–40: 23.8 vs. 23.0, *p* < 0.001; 40–65: 23.8 vs. 23.0, *p* < 0.001; >65: 23.5 vs. 22.9, *p* < 0.001; <18: 22.8 vs. 22.8, *p* = 0.442) (Table 2 and Table S2). These findings indicate that the observed time-of-day-associated variation in serum CO_2_ was substantially confounded by patient case mix.

### 3.2. False-Positive Rates of PBRTQC by Instrument and Period

**Figure 2:**
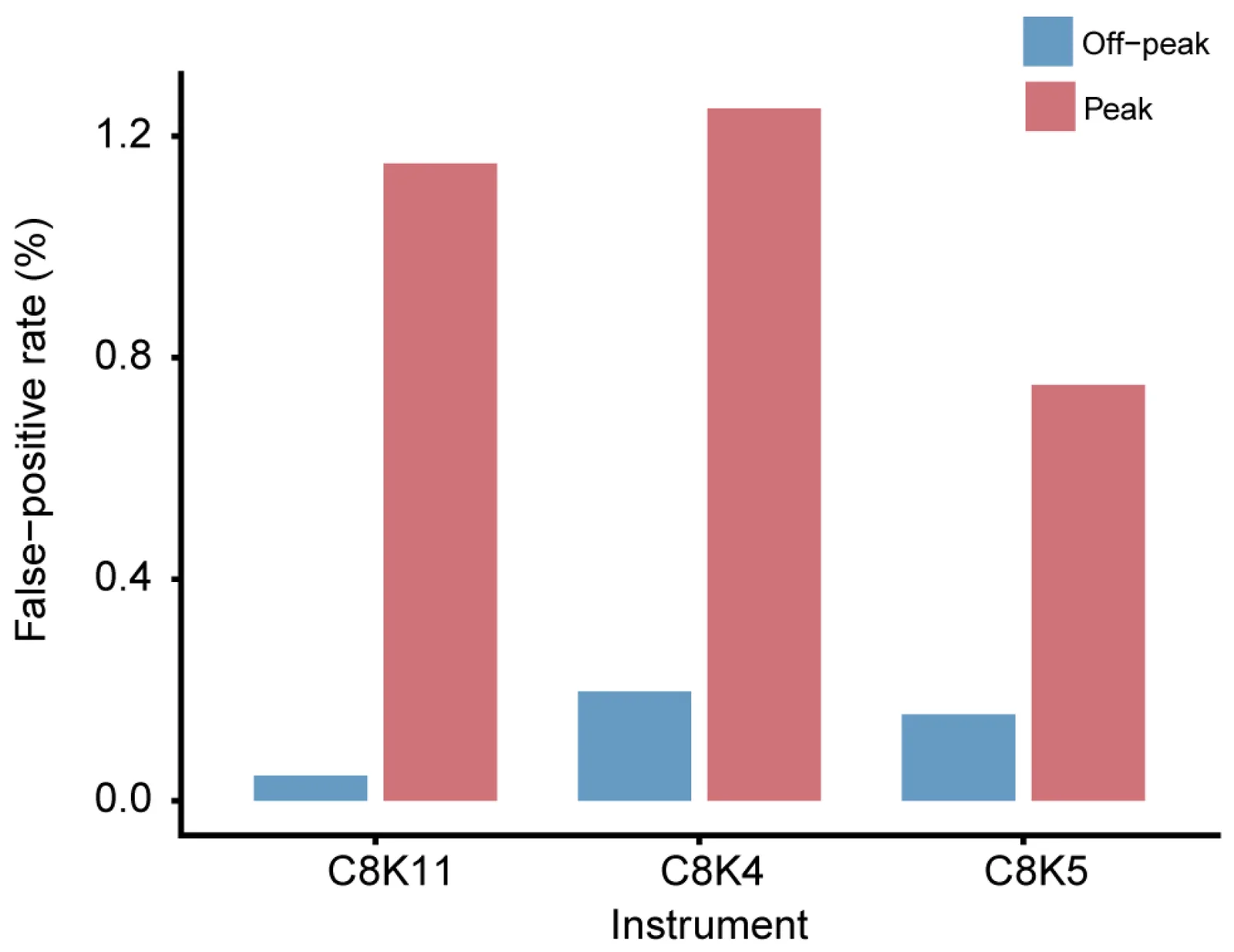
FPR was compared between peak and off-peak periods across three analyzers (C8K4, C8K5, C8K11). PBRTQC used MA with block size 15, ±1.2 SD control limits, and 5th–95th percentile winsorization. All devices showed higher FPR in peak hours.

The false-positive rates (FPR) of the PBRTQC procedure in the peak and off-peak periods for each instrument are shown in Figure 2. All three instruments had relatively high FPR in the peak hour. C8K4 had the highest peak-period FPR of 1.250%, compared with 0.197% during off-peak hours (6.3-fold increase). C8K5 showed a peak FPR of 0.751% versus 0.156% off-peak (4.8-fold increase). C8K11 had a peak FPR of 1.151%, with the largest relative increase of 25.6 times compared with its off-peak FPR of 0.045%. The detailed FPR values for each instrument are provided in Supplementary Table S4. When data from all three analyzers were combined, the overall peak-period FPR under unified limits was 0.955%, compared with 0.062% during off-peak periods.

### 3.3. Optimization of PBRTQC Using Time-Stratified Control Limits

To address the elevated false-positive rate during peak hours, we compared the traditional unified control limits (based on all data) with a time-stratified method using period-specific control limits (Table 3). As shown in Table 3, the time-stratified approach extended the control limits during the peak period (19.9–27.2 mmol/L compared with 19.8–26.4 mmol/L for the unified method), and the control limits for the off-peak period were almost the same (19.7–26.3 mmol/L). Thus, the peak-period FPR dropped from 0.955% to 0.069%, a 92.8% reduction. Off-peak FPR was still 0.062%. These results suggest that time-stratified PBRTQC parameters can reduce the excess false-positive rate associated with time-of-day-associated variation without affecting off-peak performance.

### 3.4. Error Detection Performance

**Figure 3:**
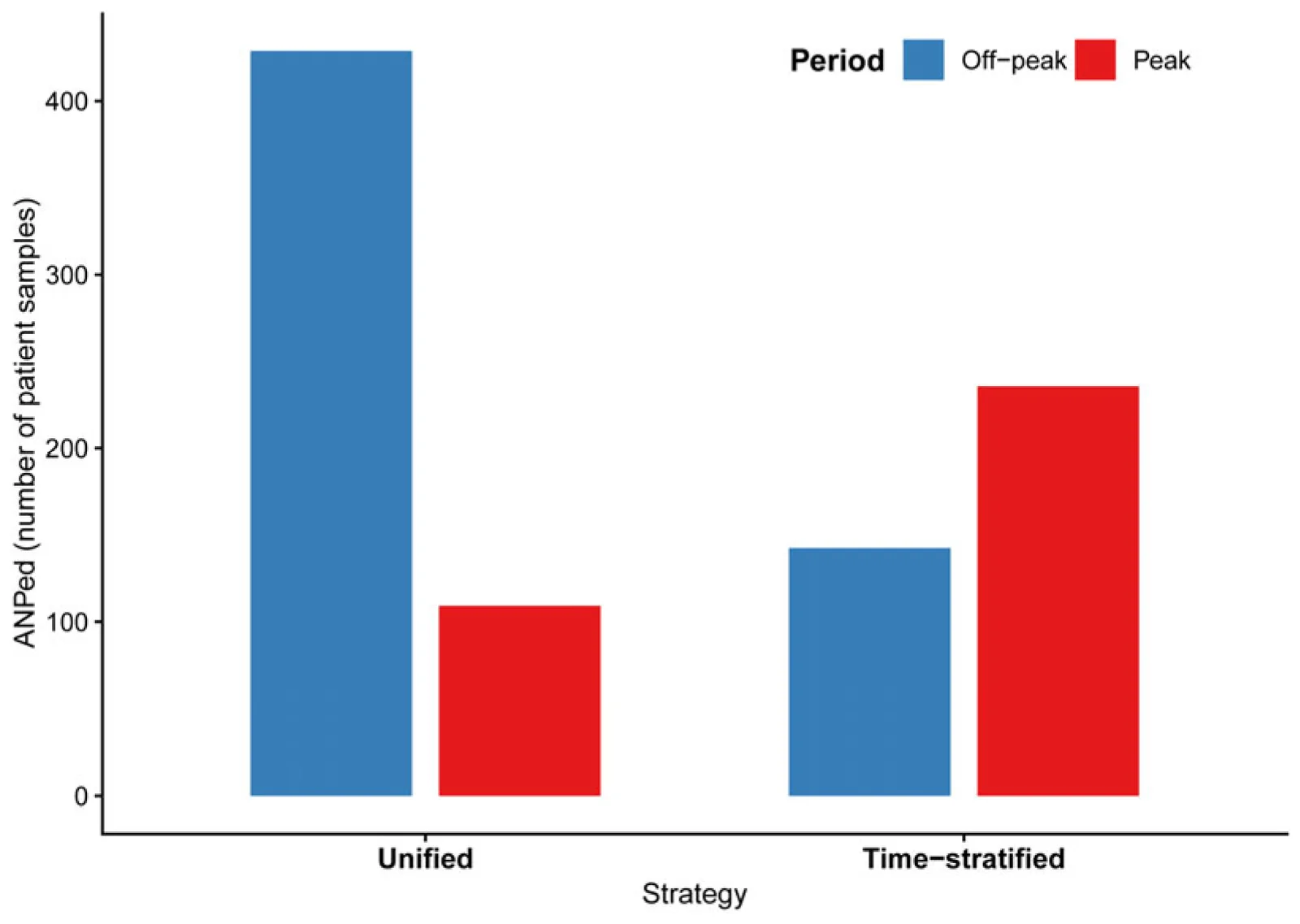
Bar chart comparing ANPed between peak (10:00–13:00) and off-peak periods under unified and time-stratified control limits. PBRTQC parameters: block size 15, ±1.2 SD control limits, 5th–95th percentile winsorization. Ped was 100% in all groups. Red = peak, blue = off-peak.

The ability of PBRTQC to detect a clinically relevant 12% constant bias was evaluated under both unified and time-stratified control limits. As shown in Figure 3 and Table S5, the probability of error detection (Ped) was 100% under both strategies. Under unified control limits, the peak-period ANPed was 109.2, compared with 428.9 during off-peak periods. Under time-stratified control limits, the corresponding ANPed values were 235.6 (peak) and 142.6 (off-peak). The difference in ANPed between peak and off-peak periods was attenuated under time-stratified control limits.

### 3.5. Error Detection Across Bias Magnitudes

**Figure 4:**
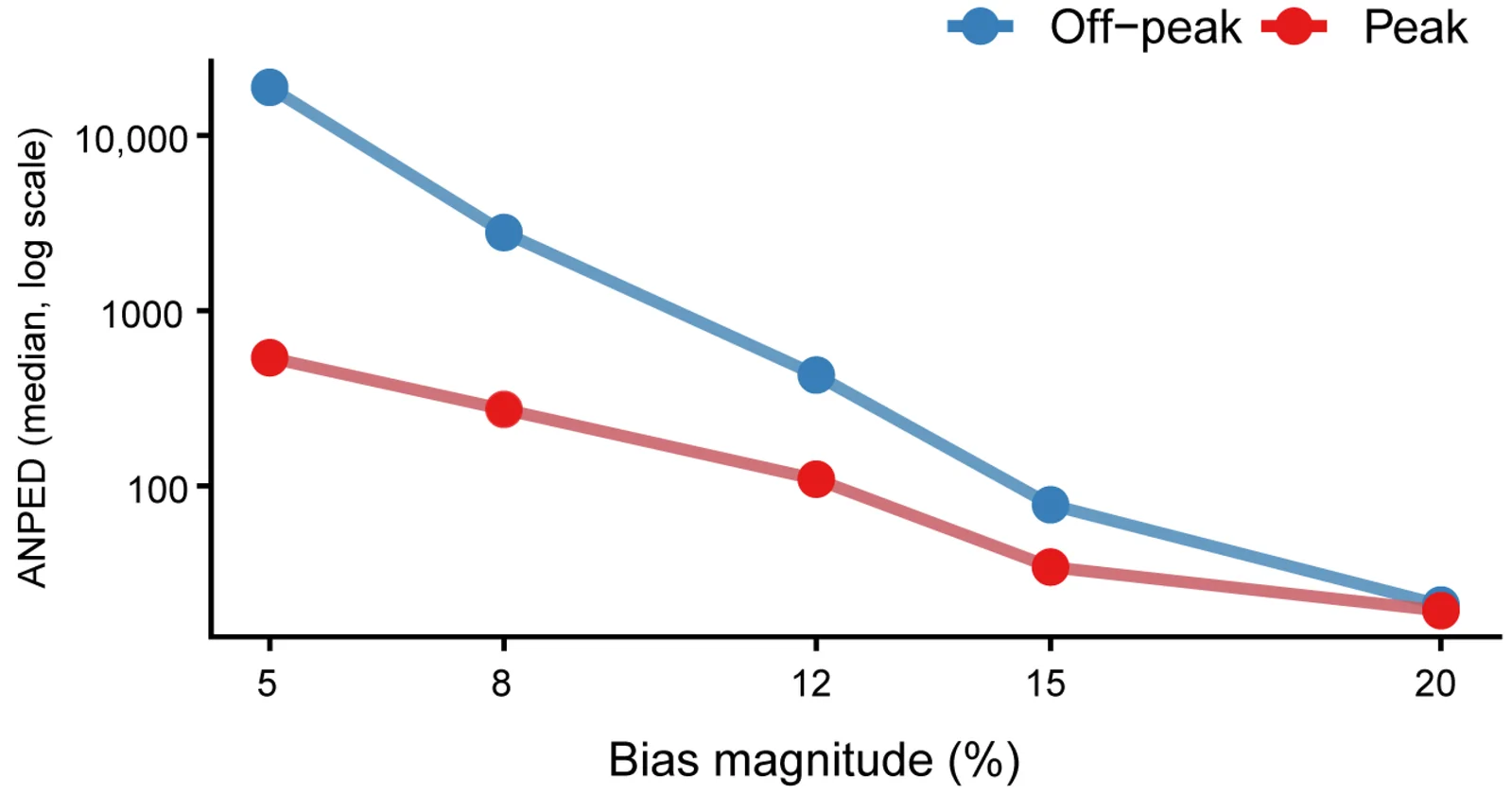
Line chart showing ANPed as a function of bias magnitude (5% to 20%) on a log scale. PBRTQC parameters: block size = 15, control limits = ±1.2 SD, winsorization limits = 5th–95th percentiles. Red = peak period, blue = off-peak period. Numerical values are provided in Table S6.

To evaluate PBRTQC sensitivity across different error magnitudes, we simulated constant biases ranging from 5% to 20% (Figure 4). Of note, for small biases of 5–8%, PBRTQC achieved 100% detection probability during the peak period, compared with only 85–90% during the off-peak period. The peak period also exhibited substantially lower ANPed at these small bias levels. For biases of 12% and above, both periods achieved 100% error detection, although the peak period still required fewer affected patient samples. Detailed ANPed values are provided in Table S6. These findings suggest that time-of-day-associated variation in the patient-result distribution altered PBRTQC sensitivity and led to earlier error detection, especially for small analytical biases.

### 3.6. Exploratory Analysis of Clinical Classification Shifts

An exploratory simulated category-transition analysis was conducted to examine the potential clinical impact of time-of-day-associated variation in the patient-result distribution using 1212 emergency resuscitation room samples collected during off-peak periods (Table 4). The observed mean difference between peak and off-peak periods in this subgroup was 0.72 mmol/L, slightly higher than the 0.60 mmol/L observed in the full dataset. The baseline classification distribution of the 1212 reference samples was acidosis: 882 (72.77%); normal: 315 (25.99%); and alkalosis: 15 (1.24%). The samples were distributed evenly across the three analyzers (C8K4: 404; C8K5: 399; C8K11: 409) and across all 12 months of 2025 (range 88–132 per month). Under the simulated peak-shift scenario, 1120 samples (92.41%) remained in their original clinical categories, while 92 samples (7.59%) were reclassified. Among reclassified cases, 88 (95.6%) were acidosis cases shifted to the normal category, and 4 (4.4%) were normal cases shifted to the alkalosis category. These findings describe a shift of some samples from the acidosis category to the normal category under the simulated peak-period condition, but they should be interpreted with caution due to the exploratory and simulation-based nature of this analysis.

## 4. Discussion

Based on one year of real-world data, this study systematically investigated time-of-day-associated variation in the serum CO_2_ patient-result distribution with regard to its effect on PBRTQC. We found that the serum CO_2_ patient-result distribution shifted toward higher values during the peak period (10:00–13:00) compared with the off-peak period. Due to this time-of-day-associated variation, PBRTQC performance changed; FPR increased to 0.955%, while ANPed decreased from 429 to 109. However, as discussed below, this apparent acceleration of error detection under unified control limits was largely a consequence of the narrower effective control limits during peak hours, rather than a genuine increase in error sensitivity. In an exploratory simulated category-transition analysis, 92 of 1212 samples (7.59%) were reclassified under the simulated peak shift, with 88 acidosis-to-normal shifts and four normal-to-alkalosis shifts. Time-of-day-associated variation in patient-result distributions has previously been shown to interfere with the results of laboratory quality control, and therefore, special strategies need to be adopted to address this problem [10].

The observed time-of-day-associated difference may reflect patient case mix, preanalytical factors, workflow patterns, environmental factors, or true within-person biological variation; this study cannot distinguish among these. Temporal changes in serum CO_2_ may be associated with various patient-related and preanalytical factors, including patient case mix and laboratory workflow patterns, though the present study was not designed to identify their specific causes [12,13]. Future studies with paired longitudinal sampling and detailed clinical information are needed to disentangle these mechanisms. Previous studies have found that time-of-day-associated variation of other analytes in serum, such as calcium, can also affect the performance of PBRTQC in the same way, and this phenomenon is often observed in clinical laboratories [14].

To the best of our knowledge, this is the first study to use a simulated category-transition approach to quantify the effect of time-of-day-associated variation in serum CO_2_ patient-result distribution on PBRTQC performance. Most of the previous PBRTQC research has concentrated on the development of algorithms, parameter optimization, and data transformation. Smith et al. emphasized the importance of a favorable underlying analyte distribution for reliable PBRTQC [15], and Bietenbeck et al. demonstrated that appropriate statistical transformation can improve detection accuracy, particularly for analytes with skewed distributions [10]. Bietenbeck et al. also noted that shifts in the patient-result distribution can lead to increased false alarms in PBRTQC, which is consistent with our observation of elevated peak-period FPR under unified control limits [10]. However, the regular effects of different times of day on PBRTQC performance have not been studied systematically. Based on our results, it can be concluded that time-of-day-associated variation in the patient-result distribution may alter both the sensitivity and specificity of the test, and this needs to be considered in actual work. This is in line with previous work of the International Federation of Clinical Chemistry and Laboratory Medicine (IFCC) PBRTQC Working Group, which pointed out that PBRTQC needs to be optimised to address analyte-specific variability [16].

It should be noted that although the exponentially weighted moving average (EWMA) algorithm is more susceptible to small biases, it had an unacceptably high false-positive rate in our first tests (peak FPR = 48.2%, off-peak FPR = 31.3%; Table S7). In line with the previous report that EWMA may be too sensitive in real-world data, it can produce an unreasonable false-positive rate (FPR) in the clinic [17]. Therefore, among the choices, the moving average (MA) method was chosen to address the high false-positive rate associated with time-of-day-associated variation and to optimize the performance of patient-based real-time quality control (PBRTQC); in the present study, we implemented a time-stratified PBRTQC strategy with period-specific control limits to improve the trade-off between sensitivity and specificity of routine laboratory monitoring [6].

Given the relatively large FPR in the peak period, we applied time-stratified PBRTQC and set different control limits for peak and off-peak times independently. Compared with the old unified strategy, time-stratified PBRTQC reduced the peak-period FPR from 0.955% to 0.069%, a 92.8% reduction, and kept the FPR during off-peak hours almost the same. As the mean and standard deviation of CO_2_ in each period were relatively stable, the observed increase in FPR was not due to a change in the spread of the data, but rather, it was a result of the corresponding change in the control limit. Thus, the time-stratified model reduced the excess false alarms caused by time-of-day-associated variation and normalized the peak-hour FPR to close to that in the off-peak period. The same method of subgroup division was also used to improve the PBRTQC performance of other analytes, such as serum calcium; that is to say, by dividing patients into different groups, false alarms and errors were reduced [14]. Recently, some research has started to consider the value of customized control limits in PBRTQC for dealing with time-of-day-associated variation in patient-result distributions, supporting our method of time-stratified control limits [15].

A high false-positive rate during peak hours can cause alarm fatigue and unnecessary laboratory testing, increasing the workload of medical staff and reducing clinical efficiency [18]. However, the apparently higher error detection sensitivity during peak hours under unified control limits should be interpreted with caution. As shown in our comparison of unified vs. time-stratified control limits, the lower ANPed during peak hours under unified limits (109.2 vs. 428.9) was not accompanied by a higher Ped (both 100%), suggesting that the earlier alarms reflected false-positive signals rather than true error detection. Under time-stratified control limits, the ANPed values became more comparable between peak and off-peak periods (235.6 vs. 142.6), providing a more accurate assessment of true error detection capability. In the exploratory simulated category-transition analysis, 88 of the 92 reclassified cases (95.6%) were acidosis-to-normal shifts, suggesting that the simulated peak shift primarily affected samples near the acidosis–normal boundary. The risk in emergency and intensive care departments is relatively high, and prompt correction of acid–base imbalance must be included in the treatment [19]. Considering that serum CO_2_ patient-result distribution may vary across days, as noted previously for other analytes, practitioners should be mindful of time-of-day-associated variation in serum CO_2_ [20]. Therefore, the primary benefit of time-stratified control limits is to reduce excess false-positive alarms while maintaining error detection capability, rather than to accelerate error detection.

There are also some limitations to the current study. First and most importantly, the observed time-of-day-associated differences in serum CO_2_ were derived from cross-sectional patient populations rather than repeated measurements from the same individuals. Because repeated measurements were available for less than 1% of records, a within-person paired analysis was not feasible. Therefore, the observed differences cannot be definitively attributed to true within-person biological variation, as they may partly reflect variations in patient case mix across different times of day (e.g., more severe cases presenting during morning hours). The marked shift in patient source across time periods (e.g., emergency department samples increasing from 9.46% off-peak to 30.99% peak, while ward samples decreased from 76.42% to 29.24%) strongly suggests that the patient case mix was a predominant driver of the observed differences. Future studies employing paired longitudinal sampling are needed to distinguish between patient-mix effects and genuine within-individual temporal variation. Second, this was a single-center study; thus, external validation at multiple hospitals is required to confirm its generalizability, as the performance of PBRTQC may differ among laboratories due to various instruments and reagent lots for different patients [6]. Third, the exploratory simulated category-transition analysis was based on simulated peak values rather than paired measurements from the same individuals; therefore, it does not provide definitive estimates of real-world reclassification rates and should be viewed as hypothesis-generating rather than confirmatory [21]. Fourth, the alternative PBRTQC algorithm EWMA was not selected as the main approach because it had a relatively high FPR in the initial tests; however, modified EWMA parameters may be explored for better performance in future studies [9,22]. Finally, the clinical outcomes at the individual patient level were not observed directly, and the actual effects of acidosis reclassification will need to be studied prospectively in future work [23]. In addition, because the peak interval was defined using the same dataset employed for PBRTQC evaluation, the reported performance differences should be regarded as descriptive. Future studies should pre-specify time windows based on independent data, perform derivation/validation splits across different time periods (e.g., first half vs. second half of the year), and conduct within-department sensitivity analyses to further disentangle patient case-mix effects from true temporal variation in analytical performance. Although there are some limitations, our results are in line with the current direction of research in recent years to address analyte variation in PBRTQC [24,25].

In summary, time-of-day-associated variation in the serum CO_2_ patient-result distribution has a notable influence on PBRTQC, revealing a distinct trade-off between sensitivity and specificity during peak hours. In the exploratory simulated category-transition analysis, most reclassified cases were acidosis-to-normal shifts (88/92, 95.6%). Time-stratified PBRTQC can reduce the false-positive rate at the peak time to some extent and maintain stable off-peak performance. In accordance with recent experimental recommendations for optimizing PBRTQC, our findings support the use of time-stratified control limits to reduce false alarms and improve the stability of continuous quality control in the laboratory [23,26]. Future multi-center studies are needed to validate this approach and assess its generalizability, as recommended by the IFCC PBRTQC Working Group [4].

## Figures and Tables

**Table 1:** Distribution of clinical department between peak and off-peak periods.

Department	Off-Peak N	Peak N	Total N	Peak %	Off-Peak %
Emergency	12,806	10,248	23,054	30.99	9.46
ICU	7886	883	8769	2.67	5.82
Outpatient	11,233	12,270	23,503	37.10	8.30
Ward	103,471	9668	113,139	29.24	76.42
Total	135,396	33,069	168,465	100	100

Other departments (*n* = 492, 0.29%) were excluded from the chi-squared test due to small sample size.

**Table 2:** Age-stratified comparison of the serum CO_2_ patient-result distribution between peak and off-peak periods.

Age Group	Off-Peak MEAN (SD)	Peak Mean (SD)	Mean Difference	*p* Value
<18	22.8 (2.67)	22.8 (2.87)	0.0	0.442
18–40	23.0 (2.60)	23.8 (2.85)	0.8	<0.001
40–65	23.0 (2.57)	23.8 (2.92)	0.8	<0.001
>65	22.9 (2.93)	23.5 (3.20)	0.6	<0.001

**Table 3:** Comparison of conventional and time-stratified PBRTQC performance.

Strategy	Period	Mean (mmol/L)	SD	Control Limits (mmol/L)	FPR (%)
Conventional (unified)	Peak	23.6	3.01	19.8–26.4	0.955
Conventional (unified)	Off-peak	23.0	2.71	19.8–26.4	0.062
Time-stratified	Peak	23.6	3.01	19.9–27.2	0.069
Time-stratified	Off-peak	23.0	2.71	19.7–26.3	0.062

Conventional control limits were computed from the entire dataset.

**Table 4:** Baseline characteristics and simulated category transitions of the emergency resuscitation room off-peak reference set (*n* = 1212).

(Panel A. Baseline characteristics)					
Characteristic		Value			
Total samples, n		1212			
Period		Off-peak			
Department		Emergency resuscitation room			
Year		2025 (January–December)			
Analyzer distribution, n (%)					
C8K4		404 (33.33)			
C8K5		399 (32.92)			
C8K11		409 (33.75)			
Baseline classification, n (%)					
Acidosis (CO_2_ < 22 mmol/L)		882 (72.77)			
Normal (CO_2_ 22–29 mmol/L)		315 (25.99)			
Alkalosis (CO_2_ > 29 mmol/L)		15 (1.24)			
Sex, n (%)					
Female		418 (34.49)			
Male		794 (65.51)			
Age, mean (SD), years		62.48 (19.27)			
(Panel B. Simulated 3 × 3 category-transition matrix under peak-period shift (+0.72 mmol/L)					
Baseline (Off-Peak)	Simulated Acidosis		Simulated Normal	Simulated Alkalosis	Total
Acidosis	794		88	0	882
Normal	0		311	4	315
Alkalosis	0		0	15	15
Total	794		399	19	1212

Numbers on the diagonal represent samples that remained in their original category. Off-diagonal numbers represent reclassified samples.

## Data Availability

The datasets used and analyzed during the current study are available from the corresponding author upon reasonable request.

## Supplementary Materials

The following supporting information can be downloaded at: https://www.mdpi.com/article/10.3390/diagnostics16213504/s1, Figure S1: Flow diagram of sample inclusion and exclusion; Table S1: CO_2_ measurements stratified by instrument and period; Table S2: Age-stratified comparison of the serum CO_2_ patient-result distribution between peak and off-peak periods (full version); Table S3: Sex distribution between peak and off-peak periods; Table S4: False-positive rates of PBRTQC by instrument and period; Table S5: Error detection performance by instrument for 12% constant bias; Table S6 Error detection performance by bias magnitude; Table S7: Comparision of MA and EWMA performance for CO_2_ PBRTQC.
