# Supplementary material for: Time-of-Day-Associated Variation in Serum CO2 Patient-Result Distribution and Its Impact on PBRTQC Performance
Source: Diagnostics (Basel). 2026 Oct 26;16(21):3504. doi: 10.3390/diagnostics16213504 (PMC13762405; doi:10.3390/diagnostics16213504)
Supplement: Supplementary file 1 [file diagnostics-16-03504-s001.zip › diagnostics-4612734-supplementary.pdf]

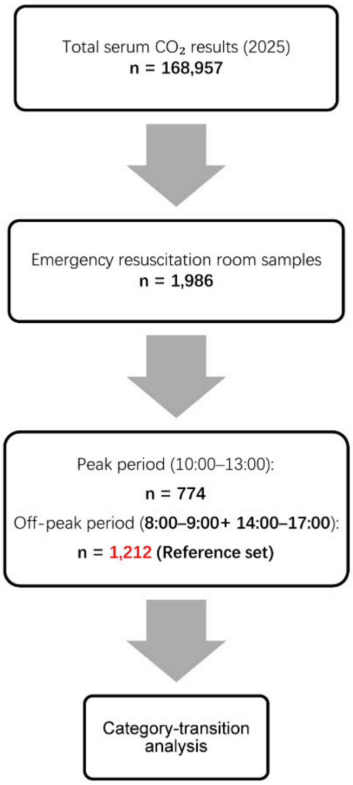

Figure S1. Flow diagram of sample inclusion and exclusion;.

Table S1. CO_2 measurements stratified by instrument and period.

Instrument	Period	n	Mean ± SD	Mean Difference (95% CI)	*p*-Value
C8K11	Peak	11,558	23.7 ± 2.98	0.70 (0.64-0.76)	<0.001
	Off-peak	46,478	23.0 ± 2.72	–	
C8K4	Peak	10,959	23.6 ± 3.06	0.60 (0.54-0.66)	<0.001
	Off-peak	44,080	23.0 ± 2.73	–	
C8K5	Peak	10,792	23.6 ± 2.99	0.70 (0.64-0.76)	<0.001
	Off-peak	44,890	22.9 ± 2.68	–	
Total	Peak	33,309	23.6 ± 3.01	0.60 (0.58-0.62)	<0.001
	Off-peak	135,648	23.0 ± 2.71	–	

Table S2. Age-stratified comparison of the serum CO_2 patient-result distribution between peak and off-peak periods (full version).

Age Group	Period	N	Mean	SD	Median	*p*-Value
<18	Off-peak	6,654	22.8	2.67	22.9	0.442
<18	Peak	2,974	22.8	2.87	23.0	—
18–40	Off-peak	17,487	23.0	2.60	23.1	<0.001
18–40	Peak	5,691	23.8	2.85	24.0	—

40–65	Off-peak	64,589	23.0	2.57	23.1	<0.001
40–65	Peak	13,720	23.8	2.92	24.0	—
>65	Off-peak	46,918	22.9	2.93	23.0	<0.001
>65	Peak	10,924	23.5	3.20	23.7	—

p values are from Welch's t-test comparing peak vs. off-peak within each age group.

Table S3. Sex distribution between peak and off-peak periods.

**Sex**	**Off-peak N**	**Peak N**	**Total N**	**Peak %**	**Off-Peak %**
Female	56,687	14,657	71,344	44.0	41.8
Male	78,961	18,652	97,613	56.0	58.2
**Total**	**135,648**	**33,309**	**168,957**	**100**	**100**

χ^2=53.61, df=1, P=2.445e-13. Percentages are column percentages within each period.

Table S4. False-positive rates of PBRTQC by instrument and period.

**Instrument**	**Off-Peak FPR (%)**	**Peak FPR (%)**	**Ratio (Peak/Off-Peak)**
C8K4	0.197	1.250	6.3
C8K5	0.156	0.751	4.8
C8K11	0.045	1.151	25.6
Total	0.062	0.955	15.4

PBRTQC parameters: moving average algorithm, block size = 15, control limits = ±1.2 SD, winsorization limits = 5th-95th percentiles. FPR: false-positive rate..

Table S5. Error detection performance by instrument for 12% constant bias.

**Strategy**	**Period**	**Control Limits (mmol/L)**	**Ped (%)**	**ANPed**
Conventional (unified)	Peak	19.8–26.4	100	109.2
Conventional (unified)	Off-peak	19.8–26.4	100	428.9
Time-stratified	Peak	19.9–27.2	100	235.6
Time-stratified	Off-peak	19.7–26.3	100	142.6

PBRTQC parameters: moving average algorithm, block size = 15, control limits = ±1.2 SD, winsorization limits = 5th-95th percentiles. Ped was 100% for all instruments and periods. ANPed: average number of patient samples affected before error detection.

Table S6. Error detection performance by bias magnitude.

Bias (%)	Period	Ped (%)	ANPed
5	Off-peak	85	18808.2
5	Peak	100	538.5
8	Off-peak	90	2789.6
8	Peak	100	273.2
12	Off-peak	100	428.9
12	Peak	100	109.2
15	Off-peak	100	78.0
15	Peak	100	34.4
20	Off-peak	100	20.9
20	Peak	100	19.4

Table S7. Comparision of MA and EWMA performance for CO_2 PBRTQC.

Algorithm	Period	FPR (%)	ANPed	Ped (%)
MA	Peak	0.955	109	100
MA	Off-peak	0.062	429	100
EWMA	Peak	48.18	5.0	100
EWMA	Off-peak	31.26	5.4	100

PBRTQC parameters: moving average algorithm, block size = 15, control limits = ±1.2 SD, winsorization limits = 5th-95th percentiles. EWMA weighting factor $\lambda = 0.125$ (equivalent to block size 15). FPR: false-positive rate; ANPed: average number of patient samples affected before error detection; Ped: probability of error detection (12% constant bias)..
